# Human Leukocyte Antigen Fine-Mapping and Correlation Analysis of Han and Minority Leprosy Patients in Southern China

**DOI:** 10.3389/fgene.2022.888361

**Published:** 2022-06-13

**Authors:** Zhuo Li, Yirui Wang, Wencheng Fan, Chang Zhang, Hao Liu, Ruixue Zhang, Lu Cao, Qi Zhen, Weiwei Chen, Yafen Yu, Bao Li, Yiwen Mao, Yuanming Bai, Daiyue Wang, Sihan Luo, Yuanyuan Li, Qin Qin, Huiyao Ge, Liang Yong, Xia Hu, Yanxia Yu, Liangdan Sun

**Affiliations:** ^1^ Department of Dermatology, No. 1 Hospital, Anhui Medical University, Hefei, China; ^2^ Institute of Dermatology, Anhui Medical University, Hefei, China; ^3^ Key Laboratory of Dermatology, Anhui Medical University, Ministry of Education, Hefei, China; ^4^ Anhui Provincial Institute of Translational Medicine, Hefei, China; ^5^ Inflammation and Immune Mediated Diseases Laboratory of Anhui Province, Hefei, China

**Keywords:** leprosy, fine-mapping analysis, HLA, correlation analysis (CA), CNV (copy number variant), susceptibility - complex

## Abstract

**Backround:** Leprosy is very prevalent in many populations around the world, which is well known that both alleles for human leukocyte antigen (HLA) as well as single nucleotide polymorphisms (SNPs) in the HLA region are common in leprosy patients. Previous studies have identified leprosy-associated susceptibility genes that explain only part of disease risk and heritability. In view of the complicated characteristics of the major histocompatibility complex (MHC) region, this study aimed to explore the development and variation of HLA in leprosy and its possible mechanism.

**Methods:** Previous genome-wide association data were extracted from Han and minority populations in southern China for HLA fine-mapping studies. Insertion and deletion (INDEL), SNP, and copy number variation (CNV) imputation were determined by using the Thousand People Database (1KGP Phase 3 Dataset) as a reference panel. The HAN-MHC database was used to input the HLA classical alleles and amino acids in the MHC region, and further step-regression analysis was performed to analyze independent variation signals associated with leprosy.

**Results:** The most significant locus rs75324027 (the same locus as rs602875 in the HLA-DR region) [*p* = 7.49E-09, OR= 0.62, 95%,CI: 0.52–0.73] in the intergene region between HLA-DQA1 and HLA-DRB1 was related with leprosy in M-S(Han leprosy patients in south China)disease. In M-SM (Leprosy patients of ethnic minorities in south China)disease, one of the most significant loci of the HLA-DQB1 gene was 6-32626438-A-T (*p* = 4.49E-08, OR = 0.36, 95%,CI: 0.25–0.52). Therefore, rs75324027 is a locus in M-S disease, and 6-32626438-a-T may be a new locus in M-SM disease. The interaction between 6 and 32626438-A-T and RS75324027 was analyzed, and A significant interaction relationship was found. In the optimal model, the accuracy of prediction was 0.5974, cross-validation Consistency:10, *p* = 0.0107.

**Conclusion:** In conclusion, this study is the first to assess the association between HLA and leprosy susceptibility in Han and other minority populations in southern China using the Thousand Population database and the Han MHC database. In addition, our analysis validated the previously reported locus rs602875 in the HLA-DR region and for the first time identified an unreported independent locus in leprosy among ethnic minorities in southern China.

## Introduction

Leprosy, also known as Hansen’s disease, is a curable chronic granulomatous major infection disorder affecting the peripheral nerves and skin; it is caused by the obligate intracellular pathogen *Mycobacterium leprae*, which is still prevalent in more than 140 countries worldwide (Wong et al., 2010). Although leprosy was declared an “eliminated” global public health issue by the World Health Organization in 2000, approximately 200,000 new cases were reported globally in 2017. Extensive migration could take the disease to nonendemic areas, so leprosy remains a serious public health problem, especially in India and China. In China (WHO, 2010), around 1,600 new cases of leprosy are discovered each year, with a prevalence rate of 0.450/100,000 (Zhang et al., 2005) (Zhang F. R. et al., 2009) (Maymone et al., 2020). Our understanding of how this bacterium causes disease and interacts with human hosts is limited by the inability to grow it *in vitro* (Wong et al., 2010).

Human genes are strong factors that influence a person’s susceptibility to leprosy, with more than 30 loci across the genome connected with a leprosy phenotype (Fava et al., 2020). Genome-wide association studies have ascertained single nucleotide polymorphisms (SNPs) within the major histocompatibility complex (MHC) on chromosome 6p21 as the most prominent inherited variant connected with leprosy (Wong et al., 2010) (Zhang F. R. et al., 2009) (Liu et al., 2015) (Wang et al., 2016) (Liu et al., 2017). MHC regions contain hundreds of genes, including MHC I and II regions for the classic human leukocyte antigen (HLA) genes. These genes encode transmembrane receptors that deliver short antigenic peptides to T cells. For class I molecules, short antigenic peptides are provided to natural killer cells and specialized cells of the monocyte lineage.

However, twin studies as well as familial aggregation and separation analysis studies have shown that the genetic factors of parasitifers play a vital role in susceptibility to leprosy, and their heritability is estimated to be as high as 57%; thus, genetic studies could contribute to the understanding of *M. leprae’s* immunity and provide insights into host–pathogen relationships (Wong et al., 2010) (Todd et al., 1990) (Abel et al., 1995) (Shields et al., 1987) (Abel and Demenais, 1988). The key role of the HLA region in regulating the immune response makes it the most promising genomic candidate. HLA-DRB1*15 has been identified as one of the most remarkable risk alleles in a genome-wide association study about leprosy in China. In addition, studies have also linked a haplotype of the HLA allele to susceptibility to leprosy (Singh et al., 2007) (Gorodezky et al., 2004). However, association analysis of HLA genes is challenging because of difficulties in high-resolution allele typing and complex linkage imbalance patterns among HLA alleles. On account of the complicated linkage imbalance and haplotype structure of HLA alleles, fine-mapping of HLA and non-HLA causal mutations throughout the MHC region is challenging. Although some loci have been proven to be connected with susceptibility to leprosy, few candidate loci have shown independent replication. Moreover, current research still does not explain the majority of phenotypic variation. A copy number variation (CNV) is a configurational variation that influences the copy number. For instance, numerous copies and deletions. CNV affects about 12% of the human genome (Redon et al., 2006). In general, CNVs are connected with multiple illnesses through a variety of molecular mechanisms, for example, Mendelian diseases, autoimmunity, and HIV susceptibility (Zhang F. et al., 2009). In addition, regarding the reliability of HLA region CNV inference, in fact, an article on this method has been published (Zhen et al., 2022), which evaluates the accuracy of CNV inference, and the accuracy of HLA-DRB5 reaches 99.43%.

To the best of our knowledge, the relationship between the HLA CNV and leprosy susceptibility is unclear. To identify more MHC loci, including CNVs, SNPs, HLA alleles, and amino acid polymorphisms, associated with leprosy in Han Chinese and ethnic minority populations in southern China, we extracted MHC data from genome-wide association studies including 799 leprosy patients and 987 controls. HLA imputation was performed based on a large Han-MHC reference panel.

## Materials and Methods

### Study Participants

We used data from a previous genome-wide association study performed in a Chinese population (Liu et al., 2015) (Wang et al., 2016). In summary, leprosy samples were collected from dermatological clinics in various hospitals, and all cases were diagnosed by at least two specialists. The control group was selected as individuals with no personal history of leprosy, autoimmune disease, or systemic disease, or a family history of leprosy (in first-, second-, or third-degree relatives). Patients and controls were matched based on their race and geographic area from which they were recruited. The subjects were homogeneous, with no systematic bias or underlying demographic stratification. The study was approved by the ethics committee of the local institution and carried out in accordance with the Declaration of Helsinki. Informed consent was obtained from all subjects or their family members. All participants were from southern China.

### Data Quality Control

For each data chip, sample and site quality controls were performed. For sample quality control, samples with a locus deletion rate of >10% were filtered out. The sites were qualitatively controlled according to the following conditions: 1) The sites that could be transformed into the plus strand of HG19 were retained; 2) The loci with a typing rate of >90% were retained; 3) The loci with a minor allele frequency of >0.01 were retained; Hardy–Weinberg equilibrium was retained at the sites with a minor allele frequency of >0.001.

### Quality Control After MHC Locus Filling

After the inference was completed, all loci were qualitatively controlled. For CNV, we retained the following the sites: 1) those with a deletion rate of <10%; 2) those with a minimum allele frequency of the locus of >0.01; and 3) those with Hardy–Weinberg equilibrium for the locus of >0.001. *R* ^2^ > 0.5 for the locus was inferred.

For SNPs, we retained the following sites: 1) those with a deletion rate of <10%; 2) those with a minimum allele frequency of the locus of >0.01; and 3) those with Hardy–Weinberg equilibrium for the locus of >0.001. *R* ^2^ > 0.9 for the locus was inferred.

For the HLA types and amino acids, we retained the following sites: 1) those with a deletion rate of <10%; 2) those with a minimum allele frequency of the locus of >0.01; and 3) those with Hardy–Weinberg equilibrium for the locus of >0.001. *R* ^2^ > 0.7 for the locus was inferred.

### Correlation Analysis of the MHC Region and Conditional Regression Analysis

We used the logistic regression model in Plink 1.9 (https://www.cog-genomics.org/plink2) to conduct association analysis for each disease as well as used sex correction for diseases with complete sex information. For disease-related candidate loci, stepwise conditional regression was used for the analysis; while for each conditional regression, independently associated loci were used as covariates in the regression model until no significant loci were found. When the most significant locus was a SNP, the HLA type, amino acid, or CNV with strong linkage disequilibrium in the gene of the locus was selected for conditional regression analysis; otherwise, the SNP was selected as the covariable for conditional regression analysis.

### Basic Analysis

In this study, we used the Thousand People Database (1KGP Phase 3 Dataset) as the reference database and Beagle 4.1 (Browning and Browning, 2007) as the inference software to fill the SNPs, INDELs, and CNVs of the MHC region (from 24 to 36 Mb on chromosome 6) for the disease data. At the same time, we used the Han Chinese MHC Database (10,689 individuals) established by Zhou et al. (2016). as the reference database and Beagle 4.1 as the inference software to fill the HLA region types, amino acids, and SNPs in the MHC region for the disease data. In order to improve the inference speed, the disease data with a large sample size were divided into samples and then calculated at the same time. The number of inference iterations was set at 10, and 4 threads were used for the calculations in order to complete the filling of SNPs and CNVs in the MHC region.

### Advanced Analysis

**FIGURE 1:**
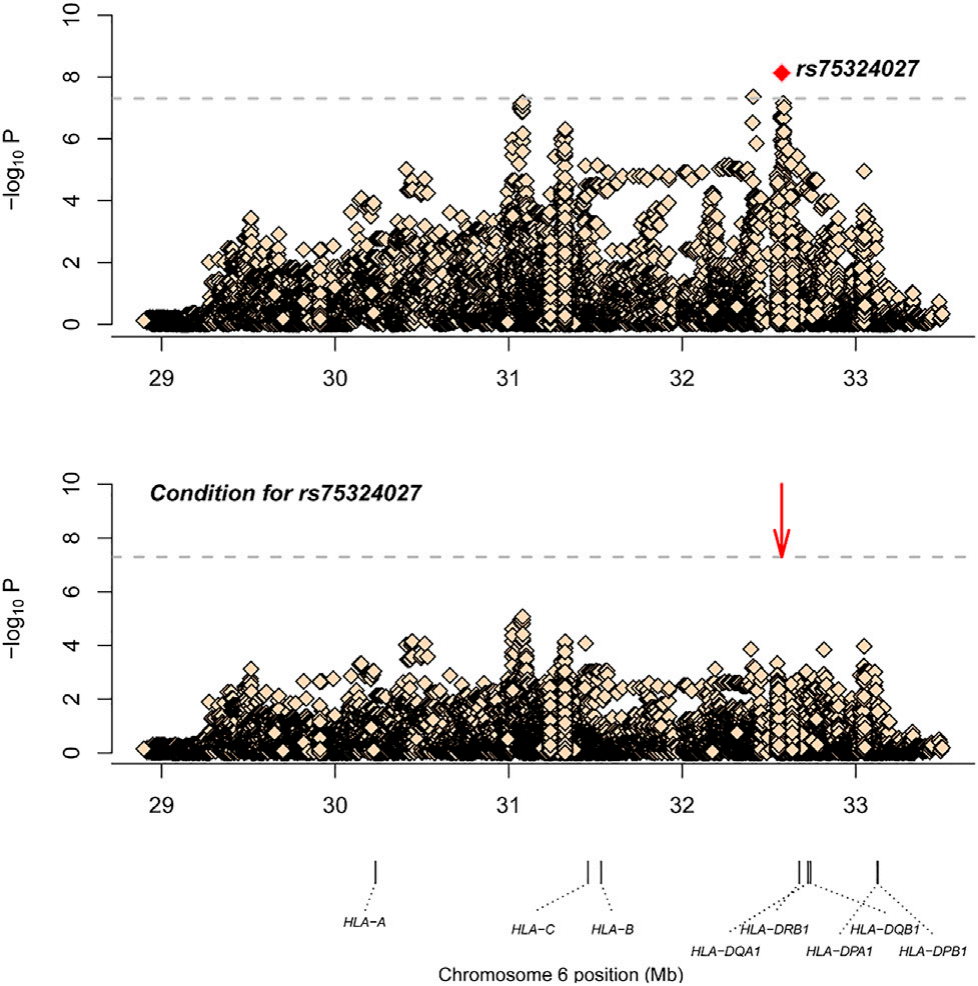
The results of M-S disease association analysis. The abscissa represents genomic loci, and the ordinate represents -log^10^(p) of the association analysis for each locus. The horizontal line represents the significance threshold *p* = 1.42 × 10^–6^. The red dots in each panel represent the sites used for conditional analysis (rs75324027).

After SNP, CNV, and HLA regional type determination, amino acid inference, and quality control for M-S disease, association analysis was conducted for 35,283 loci with 836 controls and 598 cases. The most significant locus was rs75324027 [*p* = 7.49E-09, odds ratio (OR) = 0.62, 95% confidence interval (CI): 0.52–0.73], which was located in the intergenic region between HLA-DRB1 and HLA-DQA1. After conditional analysis of the site, no site was significant (Figure 1). Wong *et al.* also have reported that SNP rs9270650, located in the intergenic region between HLA-DQA1 and HLA-DRB1, is related with leprosy (Wong et al., 2010). Therefore, rs75324027 may be a new locus.

**FIGURE 2:**
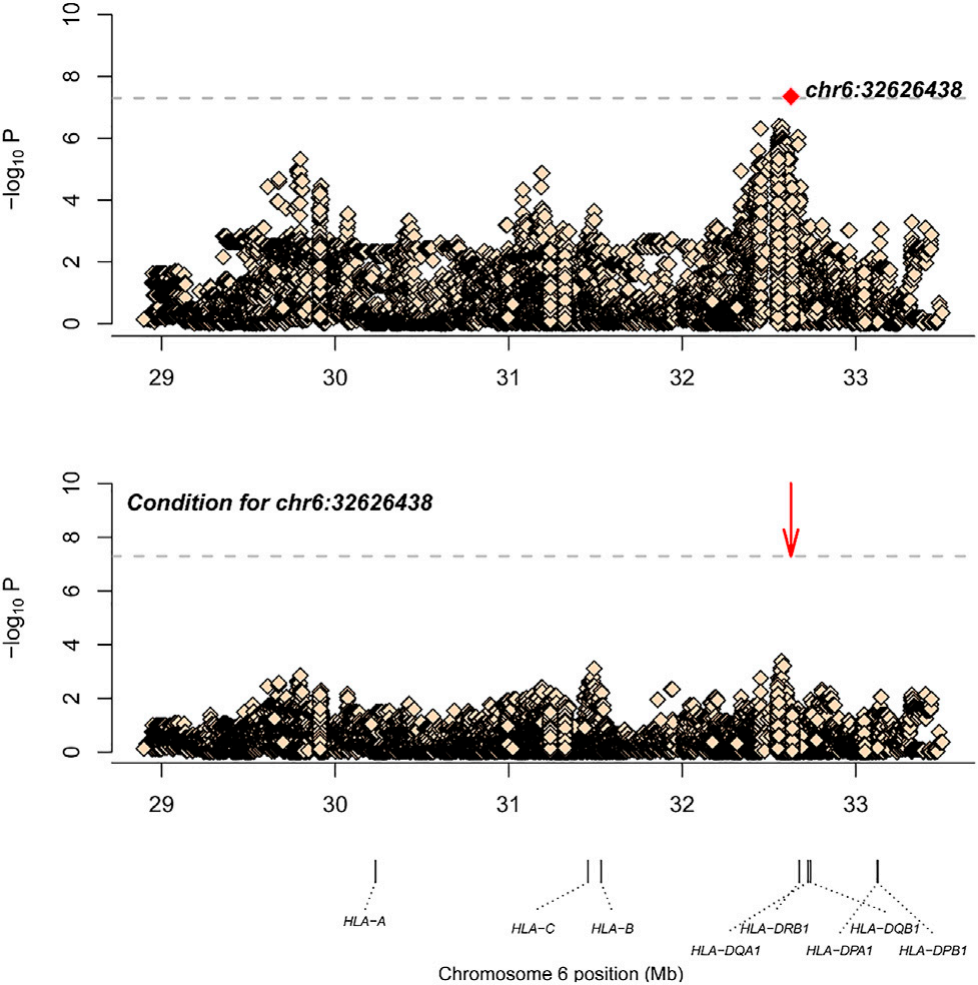
The results of M-SM disease association analysis. The abscissa represents genomic loci, and the ordinate represents -log10(p) of the association analysis for each locus. The horizontal line represents the significance threshold *p* = 1.47 × 10^–6^. The red dots in each panel represent the sites used for conditional analysis (chr6: 32626438).

Similarly, after SNP, CNV, and HLA regional type determination, amino acid inference, and quality control for M-SM disease, association analysis was conducted for 34,120 loci with 151 controls and 201 cases. The most significant locus was 6_32626438_A_T (*p* = 4.49E-08, OR = 0.36, 95%CI: 0.25–0.52), which is located in the HLA-DQB1 gene. After conditional analysis of the site, no site was significant (Figure 2). As Ohyama *et al.* (Ohyama et al., 1994) have previously reported that this gene is associated with leprosy, this locus may be a new locus.

## Results

### HLA Imputation and Association Analyses

After the data obtained through the genome-wide association studies were filtered for the set conditions, 1,456 and 1,777 SNPs from 598 patients and 836 controls from southern China and 201 patients and 151 controls from ethnic minorities in southern China were retained for quality control purposes.

When the Thousand People Database (1KGP Phase 3 Data Set) was used as the reference database and Beagle 4.1 was used as the inference software, we filled the SNPs, INDELs, and CNVs of the MHC region, respectively, for the data of each disease. In order to improve the inference speed of the CNV + SNP data, the disease data with a large sample size were divided into samples and then calculated simultaneously. In addition, while using the Han MHC Database established by Zhou et al. (2016). as the reference database, we supplemented the MHC region types, amino acids, and SNPs for the disease data, respectively (The number of inference iterations was set at 10, and 4 threads were used for the calculations.) Two of the most strongly correlated SNPs were observed in the MHC region, one of which was rs75324027 (*p* = 7.49E-09, OR = 0.62, 95%CI: 0.52–0.73) in the intergenic region between HLA-DRB1 and HLA-DQA1 in M-S disease. The other was 6-32626438-A-T, which was located in the HLA-DQB1 gene in M-SM disease (*p* = 4.49E-08, OR = 0.36, 95%CI: 0.25–0.52).

### Stepwise Logistic Regression

In order to identify independent signals of MHC regional variation, the CNV with the strongest correlation among M-S and M-SM for stepwise logistic regression was selected. The results of stepwise logistic regression analysis of leprosy-related variation are shown in Table 1 (M-S) and Table 2 (M-SM). Under the above locus conditions, none of the variants met the significance threshold. Therefore, RS75324027 and CHR6:32626438 may affect leprosy susceptibility independently in M-S and M-SM, respectively.

**TABLE 1:** Results of stepwise conditional regression analysis of M-S disease.

Step	Variant	Variant Type	Raw	Control	OR	Pvalue	Stepwise Analysis after Adjusting		
			Case				OR	Pvalue	Gene Annotation
					(95%CI)		(95%CI)		
1	Rs75324027	SNP	0.25	0.37	0.62	7.49E-09	NA	NA	Intergenic HLA-DRB1, HLA-DQA1
					(0.52–0.73)				

**TABLE 2:** Results of stepwise conditional regression analysis of M-SM disease.

Step	Variant	Variant Type	Raw	Control	OR	Pvalue	Stepwise Analysis after Adjusting		
			Case				OR	Pvalue	Gene Annotation
					(95%CI)		(95%CI)		
1	Chr6:32626438	SNP	0.19	0.38	0.36	4.49E-08	NA	NA	Intergenic HLA-DQA1
					(0.25–0.52)				

### Disease Association Analysis

#### M-S Disease Association Analysis

After SNP, CNV, and HLA regional type determination, amino acid inference, and quality control for M-S disease, correlation analysis of 35,283 loci in 836 controls and 598 cases showed that rs75324027 was the most significant locus (*p* = 7.49E-09, OR = 0.62, 95%CI: 0.52–0.73). This locus is located in the intergenomic region between HLA-DRB1 and HLA-DQA1. After conditional analysis of the site, no site was significant (Figure 1). Wong et al. (2010). also have reported that SNP rs9270650, located in the intergenic region between HLA-DQA1 and HLA-DRB1, is related with leprosy; therefore, rs75324027 may be a new independent associated locus.

#### M-SM Disease Association Analysis

After SNP, CNV, and HLA regional type determination, amino acid inference, and quality control for M-SM disease, correlation analysis of 34,120 loci in 151 controls and 201 cases showed that the most significant locus was 6-32626438-A-T (*p* = 4.49E-08, OR = 0.36, 95%CI: 0.25–0.52), which is located in the HLA-DQB1 gene. After conditional analysis of the site, no site was significant (Figure 2). Ohyama et al. (1994). also have reported that this gene is associated with leprosy, so this locus may be a new independent associated locus. As variant “6-32626438-A-T″ is located in HLA-DQB1, the relevant *p* values of the classical alleles of HLA-DQB1 measured are attached in Table 3

#### Interaction Between 6-32626438-A-T and RS75324027

By analyzing the interaction between 6-32626438-A-T and RS75324027, it is found that they show significant interaction relationship. In the optimal model, the accuracy of prediction was 0.5974, cross-validation Consistency:10, *p* = 0.0107.

## Discussion

Nearly 50 years after specific HLA antigens were first linked to disease susceptibility, a wide range of human diseases and health traits have been demonstrated to be more common in individuals carrying specific HLA alleles. The HLA region was initially identified as a major component of the graft-versus-host reaction. As research continued, HLA has been found in cancers (chronic lymphocytic leukemia, cervical cancer, and nasopharyngeal carcinoma), infectious diseases (chronic hepatitis B virus infection and chronic hepatitis C virus infection), mental nervous system diseases (Parkinson’s disease), digestive system diseases (celiac disease and ulcerative colitis), cardiovascular system diseases, endocrine system diseases (Graves’ disease and type 2 diabetes mellitus), skin diseases (psoriasis, systemic lupus erythematosus, leprosy, vitiligo, and systemic sclerosis), respiratory diseases (asthma), and other diseases of organs and systems; and the molecules and genes responsible for these conditions have been widely investigated. In view of the high linkage imbalance and high polymorphism rates of HLA genes, an international database (HLA Nomenclature) including HLA genes, alleles, coding proteins, antigens, and nomenclature was specially designed for factors of the HLA system (http://hla.alleles.org/nomenclature/naming.html).

Leprosy (MIM: 609888) is a chronic infectious and neurological disease caused by *M. leprae* that has a long history (Britton and Lockwood, 2004). Although most countries have met the World Health Organization’s leper elimination criteria (<1 per 10,000 people), there are still approximately 200,000 new cases globally each year. Previous genetic studies using family-based association analyses, candidate gene strategies, or genome-wide association studies have identified various risk loci or susceptibility genes for leprosy. These risk genes include NOD2 (MIM: 605956), PRKN [formerly PARK2 (MIM: 602544)], LRRK2 (MIM: 609007), APOE (MIM: 107741), PINK1 [formerly PARK6 (MIM: 608309)], and PARL (MIM:607858), which are involved in innate and adaptive immune systems, neural pathways, and mitochondria-related pathways. In addition, genome-wide association studies and genome-wide linkage studies have identified many risk genes that influence susceptibility to leprosy.

Although genetic research studies have shown that noncanonical MHC genes such as MICA and LTA are associated with immunity to leprosy, the major impact is attributed to the canonical class I and class II HLA genes (Tosh et al., 2006; Alcaïs et al., 2007). HLA interpolation using genome-wide association study data is an effective method for MHC fine-mapping, but there are some limitations to this approach. Through genome-wide association studies looking for susceptibility genes for leprosy, we found very significant independent related sites in southern Chinese Han patients. In addition, in southern Chinese ethnic minority patients, significant independent related sites were also found, showing that the locus of genetic variation of the gene has a significant correlation with disease. However, since the sample data analyzed in this genome-wide association study had a control group that was younger than the case group, it might be that the control group was too young to develop the disease to the clinical stage. However, in the general population, considering the lower incidence of leprosy, their relative age might have had little effect on the strength of the association.

We use the thousand Genome Project, which began in 2008 with the goal of developing common comprehensive catalogues through DNA sequencing methods. The project consists of multiple phases. Results from the initial phase of the 1,000 Genomes project were reported in 2010. The Phase I analysis, published in 2012, included the genomes of 1,092 individuals from 14 populations constructed using a combination of whole-genome coverage and exome sequencing. The first phase of the thousand Genome Project created validated haplotype maps of 38 million single nucleotide polymorphisms, 1.4 million short insertions and deletions, and more than 14,000 large deletions. The main phase of the 1,000 Genomes Project involved reconstructing the genomes of 2,504 individuals from 26 populations of European, East, South Asian, West African and American populations. The final phase of the 1,000-genome Project is phase 3, representing 2,504 samples on GRCh37.

Recently, we evaluated the relationship between HLA CNV and susceptibility to leprosy in the Han population of northern China by fine-mapping of the MHC region; we found that three SNPs may affect the pathogenesis of leprosy (Zhang et al., 2021). Previous research also has shown that genetic factors are involved in regulating the immune response against *Bacillus leprae* (Feitosa et al., 1995). Through the analysis of numerous leprosy patients in southern China, our research on HLA genes related with leprosy in a Chinese population provides evidence for the important role of HLA on leprosy incidence as well as additional support that HLA-DQA1, HLA-DRB1, and HLA-DQB1 are leprosy susceptibility alleles. For example, HLA-DRB1*15 has been shown to be an allele linked to leprosy susceptibility in Indian (Tosh et al., 2006) and Brazilian (Vanderborght et al., 2007) populations and also has been associated with an increased leprosy risk in Chinese populations (Zhang et al., 2021). The association between disease and HLA-DR-DQ loci surveyed in this study is not different from the relationship between leprosy and HLA-DRB1 identified in previous studies (Ohyama et al., 1994) (Wong et al., 2010).

Our study showed that among the SNPs associated with loci in patients with leprosy in southern China, one SNP (rs75324027) is located in the intergenomic region between non-HLA-DRB1 and HLA-DQA1, and by calculation, the LD between RS9270650 (observed by Wong et al.) and RS75324027 is 0.16. When we subsequently queried the coordinates of the locus on NCBI (https://www.ncbi.nlm.nih.gov/snp/rs75324027), it was found that the locus rs602875 was the same as the previously reported locus rs602875 in HLA-DR region, which further verified the an association with leprosy within the MHC region (SNP rs602875, next to HLADRB1) (Zhang F. R. et al., 2009). In addition, one SNP (chr6: 32626438) is located in the HLA-DQB1 gene. The SNPs among these genes are more strongly associated with patients with leprosy in southern China. Therefore, we believe that the association between leprosy and rs75324027 as well as chr6: 32626438 in patients with leprosy in southern China may involve regulatory variants. Moreover, the one newly identified loci chr6: 32626438 found in this study may be new loci associated with leprosy. Our findings greatly expand our understanding of disease susceptibility factors and suggest new biological pathways associated with leprosy.

## Data Availability

Publicly available datasets were analyzed in this study. This data can be found here: https://www.internationalgenome.org/.
