# Supplementary figures and images for: Human Leukocyte Antigen Fine-Mapping and Correlation Analysis of Han and Minority Leprosy Patients in Southern China
Source: Front Genet. 2022 Jun 13;13:888361. doi: 10.3389/fgene.2022.888361 (PMC9234480; doi:10.3389/fgene.2022.888361)

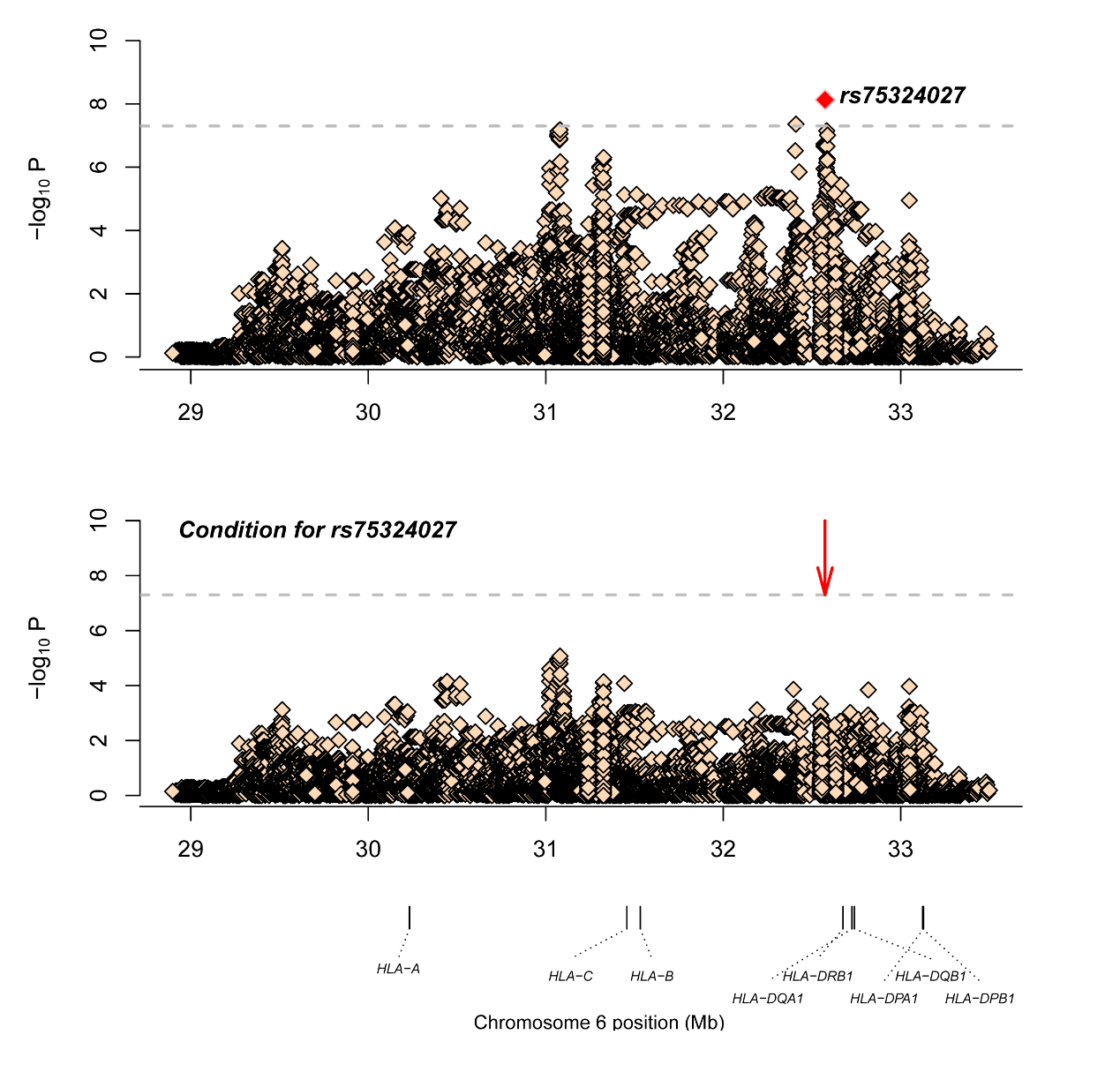

Supplement: Supplementary file 1 [file Image3.TIF]

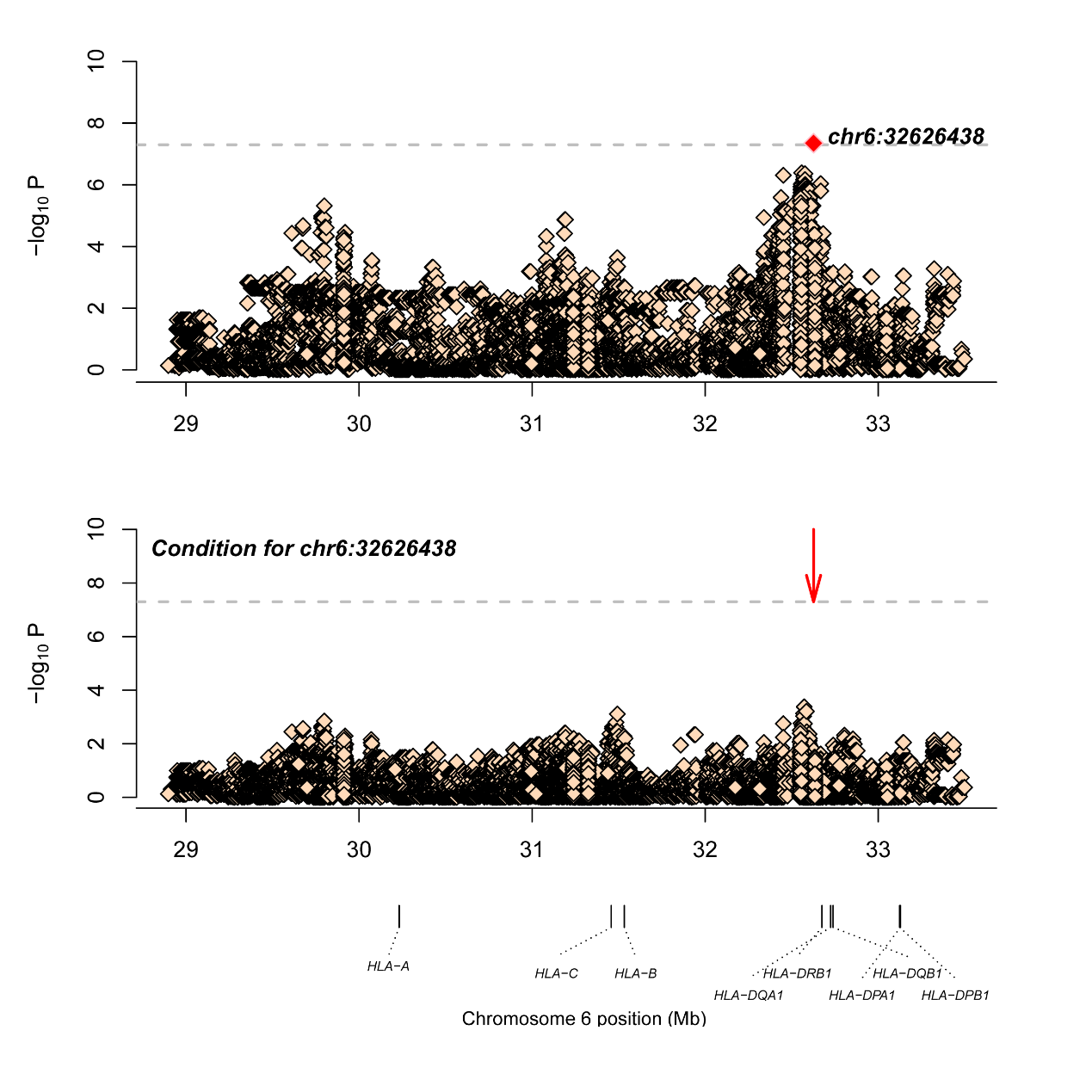

Supplement: Supplementary file 2 [file Image4.TIF]

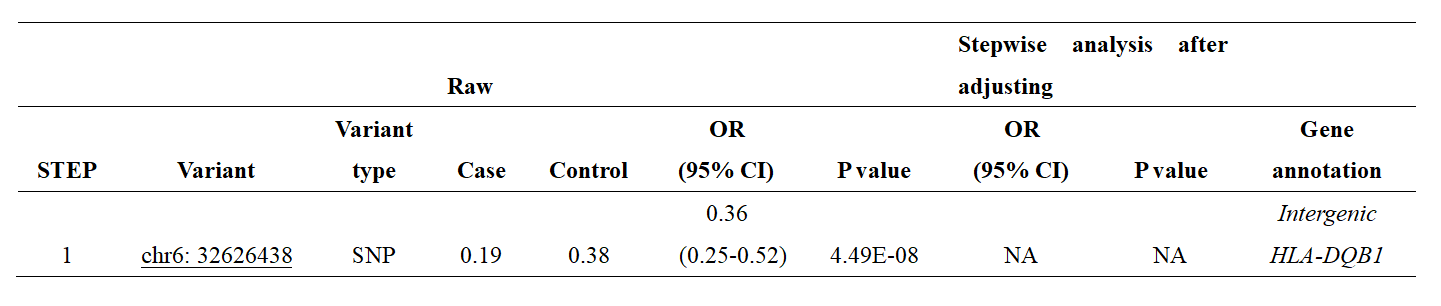

Supplement: Supplementary file 3 [file Image2.PNG]

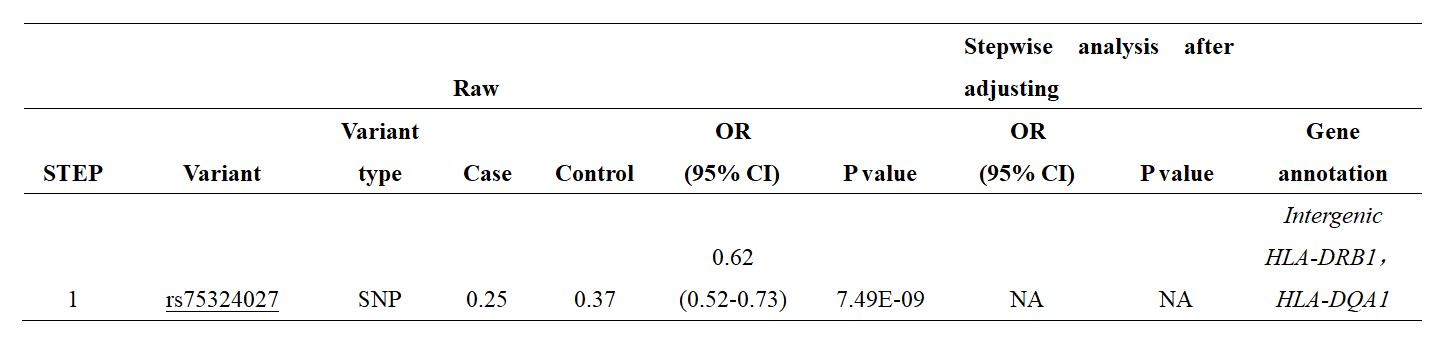

Supplement: Supplementary file 5 [file Image1.PNG]
